# A Nation-Wide Multicenter Retrospective Study of the Epidemiological, Pathological and Clinical Characteristics of Breast Cancer *In Situ* in Chinese Women in 1999 - 2008

**DOI:** 10.1371/journal.pone.0081055

**Published:** 2013-11-20

**Authors:** Yanan Kong, Lu Yang, Hailin Tang, Ning Lv, Xinhua Xie, Jing Li, Jiaoli Guo, Laisheng Li, Minqin Wu, Jie Gao, Hongjian Yang, Zhonghua Tang, Jianjun He, Bin Zhang, Hui Li, Youlin Qiao, Xiaoming Xie

**Affiliations:** 1 Department of Breast Oncology, Sun Yat-Sen University Cancer Center, State Key Laboratory of Oncology in South China, Collaborative Innovation Center for Cancer Medicine, Guangdong, China; 2 Department of Medical Imaging and Interventional Radiology, Sun Yat-Sen University Cancer Center, Guangdong, China; 3 Department of Cancer Epidemiology, Cancer Institute & Hospital, Chinese Academy of Medical Sciences & Peking Union Medical College, Peking, China; 4 Department of Breast Surgery, Zhejiang Cancer Hospital, Zhejiang, China; 5 Department of Breast-thyroid Surgery, Xiangya Sencod Hospital, Hunan, China; 6 Department of Oncosurgery, the First Affiliated Hospital of Medical College, Shanxi, China; 7 Department of Breast Surgery, Liaoning Cancer Hospital, Liaoning, China; 8 Department of Breast Surgery, the Second People’s Hospital of Sichuan Province, Sichuan, China; University of North Carolina School of Medicine, United States of America

## Abstract

**Background:**

Compared with invasive breast cancer, breast cancer *in situ* (BCIS) is seldom life threatening. However, an increasing incidence has been observed in recent years over the world. The purpose of our study is to investigate the epidemiological, clinical and pathological profiles of BCIS in Chinese women from 1999-2008.

**Methods:**

Four thousand and two hundred-eleven female breast cancer (BC) patients were enrolled in this hospital-based nation-wide and multi-center retrospective study. Patients were randomly selected from seven hospitals in seven representative geographical regions of China between 1999 and 2008. The epidemiological, clinical and pathological data were collected based on the designed case report form (CRF).

**Results:**

There were one hundred and forty-three BCIS cases in four thousand and two hundred-eleven BC patients (3.4%). The mean age at diagnosis was 48.3 years and BCIS peaked in age group 40-49 yrs (39.9%). The most common subtype was ductal carcinoma in situ (DCIS) (88.0%). 53.8% were positive for estrogen receptor (ER). Human epidermal growth factor receptor 2 (HER2) positive status was observed in 23.8% of patients. All patients underwent surgeries and 14.7% of them had breast conservation therapies (BCT) (21/143), but 41.9% accepted chemotherapy (64/143). Much less patients underwent radiotherapy (16.0%, 23/143) and among patients who had BCT, 67% accepted radiotherapy (14/21). Endocrine therapy was taken in 44.1% patients (63/143).

**Conclusions:**

The younger age of BCIS among Chinese women than Western countries and increasing number of cases pose a great challenge. BCT and endocrine therapy are under great needs.

## Introduction

Cancer In situ (CIS) is an early form of cancer, which is characteristically localized within the epithelium with the basement membrane intact and without any signs of invasion [1]. CIS, when compared with invasive cancer, often corresponds to better prognosis and is seldom life threatening. Invasive breast cancer develops from normal epithelium of the terminal duct or lobular unit through a series of events morphologically recognized as hyperplasia, atypical hyperplasia, *in situ* carcinoma, and finally malignant and invasive neoplasia [2,3]. Although most invasive cancers develop in this course, this process may be discontinuous in some other invasive cancers and is certainly not inviolate or universal. In addition, there has been evidence showing that many forms of invasive carcinoma originated from the progression of a CIS lesion [4,5]. Therefore, CIS is considered a precursor or incipient form of cancer that may, if left untreated long enough, transform into a malignant neoplasm.

Importantly, incidence rate of breast cancer in situ (BCIS) has risen dramatically over the last two decades, largely because of increased diagnosis as a result of increases in mammography screening. According to the Breast Cancer Statistics, there were about 57,650 new diagnoses of BCIS among US women in 2011, accounting for approximately 20% of all 288,130 new breast cancer cases the same year [6]. In China, due to limited cancer registries, only a few studies focus on the epidemiology of BCIS and most of them were based on small populations. So far, no nation-wide representative data of BCIS in China is available. Moreover, in recent years, great progress has been made in early detections and treatment options for BCIS. However, current data which describes these improvements and changes are almost all based on Western population [7-11]. Due to the difference of religion, race, circumstance and economic status, these data can not represent all patients over the world. Therefore, studies that could give a description of epidemiological, pathological and clinical characteristics of BCIS in other populations are of great need.

Here, we performed a nation-wide multi-center 10-year (1999-2008) retrospective clinical epidemiological study of females with BCIS in China in which patients were confirmed BCIS with pathology from 7 geographic regions across China (North, North-East, Central, South, East, North-West, and South-West). The aims of this study were to document (1) the sociodemographic characteristics and the distribution of some risk factors among Chinese female BCIS cases; (2) the clinical characteristics of female BCIS in China and (3) current treatment options for Chinese female BCIS.

## Patients and Methods

### Ethics Statement

This study was approved by the Institutional Review Board, Cancer Foundation of China. The written consent was given by the patients for their information to be stored in the hospital database and used for research.

### Study Design

This study was part of a hospital-based multi-center 10-year retrospective study of randomly selected females whose medical chart review showed primary breast cancer [12]. To study the epidemiological, pathological, clinical characteristics and treatment options of BCIS, we selected cases that were pathologically confirmed BCIS out of the data pool in these Chinese women between 1999 and 2008. 

### Selection of Hospitals and Patients

A total of 7 hospitals selected from all seven traditional China districts were involved in this study. The seven districts were north, northeast, northwest, central, east, south and southwest of China, which extend over the majority of the country and represent different levels of breast cancer burden. Each selected hospital was one of the best leading hospitals at the tertiary level and had regional referral centers providing pathology diagnosis, surgery, radiotherapy, medical oncology, and routine follow-up care for patients with breast cancer. 

Females who were pathologically confirmed primary breast cancer inpatients in one randomly selected month each from year 1999 to 2008 were enrolled. Every hospital collected patients randomly for no fewer than 50 cases in any month from March to December by an enrollment scheme. All cases collected were reviewed and patients’ information was collected based on the designed case report form (CRF) with quality control. In each selected month, if the number of inpatients were fewer than 50 in that year, additional cases from the following months were reviewed until the total number in that year reached 50. But if the number of patients exceeded 50, all cases were reviewed. To ensure that the national study was geographically representative, it was designed to include patients enrolled at sites from all seven traditional regions across China. 

### Pathology Diagnostic Criteria

Histological subtype was based on the 1981 and 2003 WHO histological classification criteria [13,14]. Staging of breast cancer was done according to the AJCC TNM staging system of year 1997 and after [15,16].

### Data Collection and Quality Control

The data were systematically collected for all enrolled patients by medical chart review as described above [12]. Briefly, it included the general information, demographic characteristics, breast cancer risk factors, clinical breast examination, imaging, pathology and treatments of the patients. All of the above information was extracted from medical charts to the designed CRF by local trained-clerks. The data were finally transferred from paper to a database (FoxPro) and double-checked to ensure accuracy.

### Sample Size

There were a total number of no fewer than 500 cases per hospital over 10 years (between 1999 and 2008) due to a minimum of 50 patients per site per year. Therefore, pooling of data across all sites in China was employed to adequately describe disease and treatment characteristics across the country.

### Data Analysis

Due to the loss of some data or too many unknowns, not all of the information in CRF was analyzed and only data that focused on the epidemiological, pathological, clinical characteristics, and treatment options were used. All data were presented based on the original CRF. 

## Results

### Trends of both primary breast cancer and BCIS cases over ten years across China

**Figure 1:**
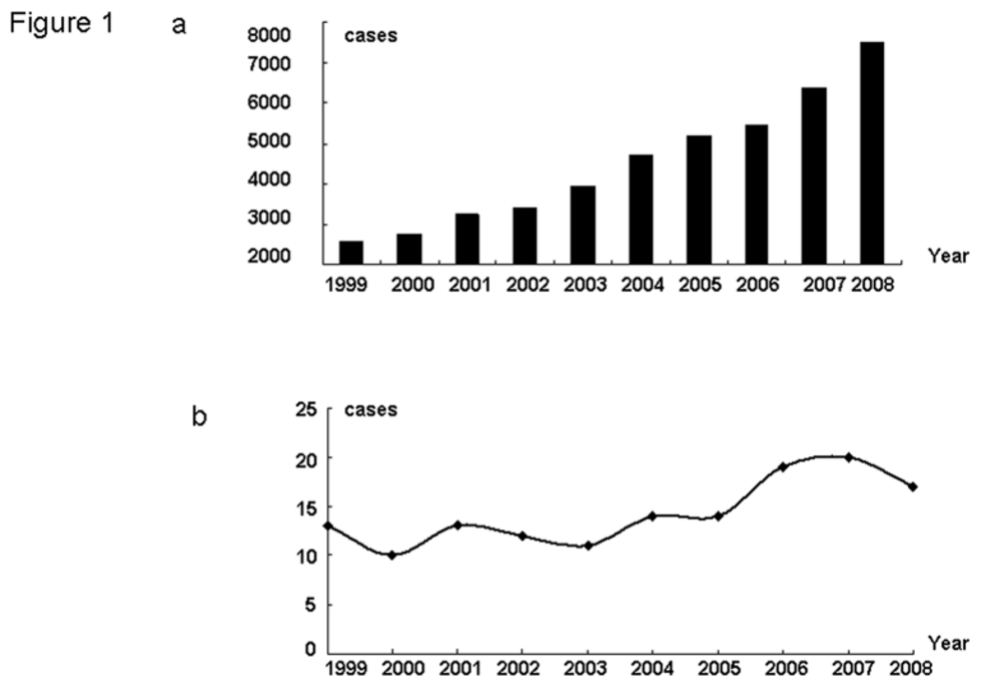
Trends of the number of inpatients of BC and BCIS over ten years. a. Numbers of Chinese breast cancer inpatients in each year between 1999-2008. b. The trend of Chinese breast cancer in situ cases in 1999-2008.

All seven selected hospitals provided the number of all their breast cancer inpatients each year, and there were a total of 45,200 patients with breast cancer during 1999- 2008. In 1999, 2,590 cases were diagnosed and under treatment, and by 2008, this number had reached 7,512 with a 2.9-fold increase (Figure 1a). Though we did not acquire all BCIS cases in the seven hospitals over ten years, cases in selected months were collected. A steady increase was observed in number of BCIS inpatients from 1999 to 2008 (Figure 1b).

### Patients Characteristics

**Figure 2:**
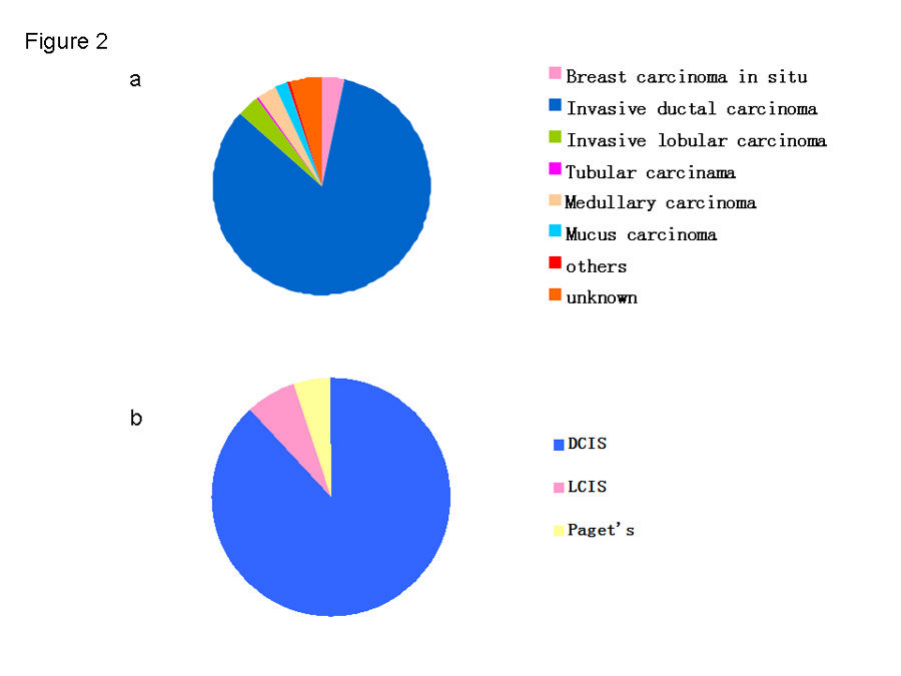
Pathological type of BC and BCIS over ten years. a. The percentage of each pathological type of BC in 19999-2008. b. The percentage of each Pathological type of BCIS in 19999-2008.

There were 143 patients diagnosed with BCIS, representing 3.4% of all 4,211 selected patients (143/4211) (Figure 2). Table 1 summarizes the general information of BCIS from seven districts during 1999-2008. The mean age at diagnosis and age range for all breast cancer patients was 48.3 years (s.d. = 11.2 yrs) and 24-80 years, respectively. The majority (69.1%) of BCIS patients were of normal Body Mass Index (BMI). Most BCIS patients were either manual or mental workers, accounting for 74.8% of all 143 patients, and housewives only presenting 3.5% of all occupations. Except for unknowns, middle school education is most common in BCIS cases. Approximately 68.5% of the cases were pre-menopausal and 31.5% were post-menopausal. The majority had been married and only two women were single. There were 94 BCIS cases with breast feeding history, accounting for 65.7% of all patients. Only 5 women had a breast cancer family history, representing only 3.5% of all cases.

**Table 1:** Epidemiological characteristics of breast carcinoma in situ in China.

Characteristics	*N*	%
Age (years)		
Mean±SD	48.28±11.24	-
Range	24~80	-
≤29	3	2.10
30-39	29	20.28
40-49	57	39.86
50-59	29	20.28
≥60	25	17.48
BMI		
Mean±SD	23.38±3.15	-
Range	16.82~31.63	-
Underweight(≤18.49)	3	2.44
Normal(18.50~24.99)	85	69.11
Overweight(25.00~29.99)	30	24.39
Obesity(≥30.00)	5	4.07
Occupation		
Housewife	5	3.50
Manual worker	56	39.16
Mental worker	51	35.66
Others	31	21.68
Education		
None	3	2.10
Primary school	8	5.59
Middle school	16	11.19
High school	9	6.29
University and above	12	8.39
Unknown	95	66.43
Menopausal status		
Pre-menopause	98	68.53
Post-menopause	45	31.47
Marital status		
Single	2	1.40
Married	138	96.50
Widowed/divorced	3	2.10
Breast feeding history		
Yes	94	65.73
No	11	7.69
Unkown	38	26.57
Breast cancer family history		
Yes	5	3.50
No	133	93.01
Unknown	5	3.50

BMI, body mass index

### The clinical and pathological characteristics of patients

Table 2 illustrates the clinical and pathologic characteristics of patients. Among all 143 BCIS cases, tumors had almost the same probability of being located on either the left or right. However, it should be noted that 8 cases were non-palpable, indicating that besides clinical physical examination, mammography and ultrasound were required for screening. Most tumors happened in the upper outer quadrant, accounting for almost half of all cases. About 55.4% of the patients had a tumor of which the size was less than 20 mm, tumors that were larger than 50 mm only present 10.7%. Over the 10 years, ductal cancer in situ (DCIS) remained the dominant pathologic subtype (88.1%). Among the 140 patients that had estrogen receptor (ER) and progesterone receptor (PR) information, fewer than 10% (8.2%) were ER positive and PR negative; 12.3% were positive with PR but negative with ER; more than half (52.5%) were both ER and PR positive and 24.6% were negative with both. Human epidermal growth factor receptor 2(HER2) information was available for 92 patients and the majority (63.0%) of them were HER2 negative.

**Table 2:** Clinical and pathological characteristics of breast carcinoma in situ in China.

Characteristics	*N*	%
Tumor location		
left	70	48.95
right	65	45.45
Non-palpable	8	5.59
Quadrant		
upper inner	21	14.69
upper outer	60	41.96
lower inner	6	4.20
lower outer	9	6.29
center	21	14.69
others	13	9.09
Tumor size		
<20mm	62	55.36
20~49mm	38	33.93
≥50mm	12	10.71
Pathology		
DCIS	126	88.11
LCIS	10	6.99
Paget's disease	7	4.90
ER/PR status		
ER+&PR+	64	52.46
ER+&PR-	10	8.20
ER-&PR+	15	12.30
ER-&PR-	30	24.59
unknown	3	2.46
HER2 status		
HER2+	34	23.78
HER2-	58	40.56
uncertain	17	11.89
unknown	5	3.50

ER, estrogen receptor; PR, progesterone receptor; HER2, human epidermal growth factor receptor-2.

### Treatment options

Treatment options for all patients were listed in Table 3. Among all BCIS cases, the majority (95.8%, 137/143) had undergone surgical procedures, with modified radical mastectomy being the predominant option (67.8%, 97/143). A minority of women (9.8%, 14/143) received breast conservative surgery. Hormonal therapy was the second most important treatment option (61.8%, 55/143) for BCIS patients in which ER/PR was positive. There were a total of 62 patients having chemotherapy, including neoadjuvant and adjuvant chemotherapy; these patients accounted for 43.4% of all cases. Only 15.4% of the patients accepted radiotherapy, and 66.7% of all those who had undergone breast conserving therapy (BCT) had radiotherapies.

**Table 3:** Treatment options for breast carcinoma in situ in China.

Options	*N*	%
Surgery		
Radical mastectomy	7	4.90
Modified radical mastectomy	97	67.83
Breast conservative surgery	14	9.79
Simple mastectomy	12	8.39
BCT+SLN biopsy	7	4.90
others	6	4.20
Radiotherapy		
No	117	81.25
RT after BCT	14	9.72
RT after Radical/Modified radical mastectomy	8	5.56
unknown	5	3.47
Chemotherapy		
No	73	49.32
Neoadjuvant chemotherapy	7	4.73
Adjuvant chemotherapy	55	37.16
Unknown	13	8.79
Endocrine therapy		
Total		
No	64	44.76
Yes	63	44.06
Unknown	16	11.19
Among ER/PR positive		
No	24	26.97
Yes	55	61.80
Unknown	10	11.24
Anong ER&PR negative		
No	24	80.00
Yes	2	6.67
Unkown	4	13.33
Among Pre-menopausal		
SERM	42	95.46
AI	2	4.54
Among Post-menopausal		
SERM	12	54.54
AI	10	45.45
Target-HER2 therapy		
No	122	85.31
Unknown	21	14.69

BCT, breast conservation therapy; SLNB, sentinel lymph node biopsy; RT, radiotherapy; SERM, selective estrogen receptor modular; AI, aromatase inhibitor.

## Discussion

So far, ours is the first geographically-representative epidemiological study of BCIS in China, with 143 patients selected from a data pool of 4211 primary breast cancer cases. This study covered a large number of sites across all seven traditional regions of China [12], making it possible for us to thoroughly access the epidemiological, clinical, pathological characteristics and managements of BCIS across the entire country. A retrospect of ten years’ time shows the trends of the number of BCIS inpatients from 1999 to 2008. It also helps us to analyze the current limitation in treatments and how to improve treatment options in China, the biggest developing country. From 1999 to 2008, both the numbers of breast cancer and BCIS inpatients have increased in China, a trend similar to the data in Western countries [6,10,17-21]. According to the statistics from the SEER program, incidence rates of BCIS in Western countries rose rapidly in the 1980s and 1990s, largely because of increased diagnosis as a result of increases in mammography screening [22-24], which may be the same reason for the current increase in China. It is important to note that since 1999, incidence rates of BCIS have stabilized in women aged 50 years and older, but continue to increase in younger women [6]. However, due to the limited data and lack of age-control groups, such results could not be acquired in our study. All patients enrolled in this study were ethnically Chinese, and their clinical characteristics were significantly different from the women in Western countries. The mean age at diagnosis was 48.3 years, and this was consistent with the findings from studies in other regions of China [25-33]. There was no difference in the mean age between breast cancer (48.7 years) and BCIS in China, both of which were around the mid-40s. The mean age was about ten years younger than the reports in Western countries, in which breast cancer clusters peak around 60-69 years [10,19]. The reasons for this difference are still unclear, but several reasons could explain it: (1) mammography screening are more frequently used among older women in Western countries. (2) older Chinese women have been less exposed to estrogen-related risk factors. (3) younger women are more aware of breast cancer due to inefficient mammography screening in China. (4) younger women were more genetically predisposed to breast cancer [12]. 

Of all BCIS cases, DCIS accounted for 88.1% and remained the dominant pathological subtype. However, in this study, DCIS had only 2.7% (113/4211) of all primary breast cancer cases, differing drastically from the data of other studies performed in Western countries. There have been reports that, before 1980s, the incidence of DCIS was low and represented ~7% of all breast cancer cases, but now, DCIS represents 15-25% of cases in countries with efficient mammography screening programs [8,34]. The increase of incidence in Western countries can easily be explained by the advent of screening mammography. Patients presented with DCIS until they had become clinically symptomatic in earlier times. There may be two reasons for low proportions observed in China. First, several studies reported the incidence of DCIS by race or ethnicity and there may be some difference between Asian and Caucasian women. Secondly, the use of mammography screening in China was not as popular as in Western countries because of lower economic levels. In recent years, there have been reports on the effectiveness of screening mammography on breast-cancer incidence especially early stage breast cancer including DCIS, suggesting that there is substantial over-diagnosis, accounting for nearly a third of all newly diagnosed breast cancers [23,35-37]. Thus, due to inefficient mammography screening programs, there may be fewer occurrences of over-diagnosis caused by mammography screening in China.

Surgery was the most common treatment in Chinese female BCIS patients, followed by hormonal therapy. Ninety-five percent of all BCIS patients underwent surgeries. Overall, modified radical mastectomy was still the most common form of surgery, accounting for 67.8% of the 143 BCIS cases. Only 15.6% of women were treated with BCT, including 4.9 percent who accepted sentinel lymph node biopsy (SLNB). This is inconsistent with studies in the USA, suggesting that a large proportion are still treated with mastectomy [19,34], in some cases combined with SLNB.

However, there was no obvious increase observed in the percentage of BCIS patients who underwent BCT in China over the ten years, which is not consistent with the data from a study showing that an increasing percentage of patients were being treated with BCT (from 10% to 70%) between 1981 to 2001 in California [34]. The less widespread use of BCT in China in that time period may be caused by three reasons: (1) Although there was no difference in overall survival (OS ) between mastectomy and BCT plus radiotherapy, most patients still choose mastectomy as the “safer option”; 2) radiotherapy was required after BCT, but due to their economic statuses, some patients could not afford the cost; (3) BCT is not only a form of surgery; it requires advanced techniques in imaging diagnosis such as MRI and radiotherapy design. Due to limited medical resources in China, it is risky to carry BCT in patients. Therefore, with more recognition of BCT by patients and development in medicine, more BCT could be performed in China, in order to increase the living quality of future patients. 

In our study, 62.2% (89/143) of patients were ER/PR positive and among these patients, 61.8% (55/89) accepted hormonal therapy. Since 1998, when U.S. Food and Drug Administration approved the use of tamoxifen to decrease the rate of recurrence of invasive breast cancer, which had been proved by several studies [38,39], the percentage of patients with DCIS treated with hormonal therapy have increased. The proportion of patients using hormonal therapy in China is much higher than that in the USA, which was reported at 14.1% [19]. Though this data was acquired in DCIS patients, due to the smaller proportion of LCIS in all BCIS, it still could demonstrate that hormonal therapy is more widespread in China than that in Western countries. However, about 40% of ER positive patients did not accept hormonal therapy, and that percentage of patients maybe expected to reduce the recurrence rate if they receive hormonal treatment. About 23.8% of all BCIS patients were HER2 positive and a similar percentage was observed in all breast cancer patients. Yet, there have been studies in Western countries reporting that pure DCIS overexpressed HER2 in approximately 45% [40]. The difference between Chinese and Caucasian population was obscure. Considering the fact that HER2 targeted therapy was not necessary for DCIS according to NCCN guidelines, this variety may not be so important for treatment choices. Compared with surgery and hormonal therapy, options for radiotherapy and chemotherapy were relatively fewer. Though there have been data demonstrating that radiotherapy after BCT could reduce the local recurrence of breast cancer [5,41,42], not all patients who underwent BCT accepted radiotherapy, showing that standard procedure should be strengthened. About 37.2% of patients had chemotherapy, which may reflect over-treatment on BCIS in China, because for *in situ* cases, chemotherapy is not the necessary choice.

This study provided a detailed description of BCIS across China. However, there are still some limitations: (1) selection bias may exist in the selected hospitals and months; (2) the number of BCIS cases was not large enough(3); There is no comparison between BCIS and invasive breast cancer. Overall, this is a representative study of BCIS in China to understand epidemiological, pathological ,clinical characteristics and therapies by over ten years’ retrospective study. The younger age of BCIS onset among Chinese women and increased number of cases pose a great challenge for the Chinese government regarding incidence control. BCT, as a surgical option safe and effective, is of great need. It is also necessary for hormonal therapy to be used more widely, in order to decrease the recurrence of invasive breast cancer. One should note that the application of chemotherapy should adhere strictly to the guidelines and its abuse should be prevented. A complete consideration of social, economic and medical factors by both government and doctors will ensure that better decisions are made regarding prevention and treatment for patients.
